# Natural language processing for the assessment of cardiovascular disease comorbidities: The cardio‐Canary comorbidity project

**DOI:** 10.1002/clc.23687

**Published:** 2021-08-04

**Authors:** Adam N. Berman, David W. Biery, Curtis Ginder, Olivia L. Hulme, Daniel Marcusa, Orly Leiva, Wanda Y. Wu, Nicholas Cardin, Jon Hainer, Deepak L. Bhatt, Marcelo F. Di Carli, Alexander Turchin, Ron Blankstein

**Affiliations:** ^1^ Cardiovascular Division, Department of Medicine, Brigham and Women's Hospital Harvard Medical School Boston Massachusetts USA; ^2^ Department of Medicine, Brigham and Women's Hospital Harvard Medical School Boston Massachusetts USA; ^3^ Division of Endocrinology, Department of Medicine, Brigham and Women's Hospital Harvard Medical School Boston Massachusetts USA; ^4^ Department of Radiology, Brigham and Women's Hospital Harvard Medical School Boston Massachusetts USA

**Keywords:** cardiovascular comorbidities, natural language processing

## Abstract

**Objective:** Accurate ascertainment of comorbidities is paramount in clinical research. While manual adjudication is labor‐intensive and expensive, the adoption of electronic health records enables computational analysis of free‐text documentation using natural language processing (NLP) tools.

**Hypothesis:** We sought to develop highly accurate NLP modules to assess for the presence of five key cardiovascular comorbidities in a large electronic health record system.

**Methods:** One‐thousand clinical notes were randomly selected from a cardiovascular registry at Mass General Brigham. Trained physicians manually adjudicated these notes for the following five diagnostic comorbidities: hypertension, dyslipidemia, diabetes, coronary artery disease, and stroke/transient ischemic attack. Using the open‐source Canary NLP system, five separate NLP modules were designed based on 800 “training‐set” notes and validated on 200 “test‐set” notes.

**Results:** Across the five NLP modules, the sentence‐level and note‐level sensitivity, specificity, and positive predictive value was always greater than 85% and was most often greater than 90%. Accuracy tended to be highest for conditions with greater diagnostic clarity (e.g. diabetes and hypertension) and slightly lower for conditions whose greater diagnostic challenges (e.g. myocardial infarction and embolic stroke) may lead to less definitive documentation.

**Conclusion:** We designed five open‐source and highly accurate NLP modules that can be used to assess for the presence of important cardiovascular comorbidities in free‐text health records. These modules have been placed in the public domain and can be used for clinical research, trial recruitment and population management at any institution as well as serve as the basis for further development of cardiovascular NLP tools.

## 1 INTRODUCTION

Extracting and accurately categorizing medical comorbidities is paramount in clinical research.^1^ The traditional approach to identification of comorbidities using manual adjudication is labor‐intensive and expensive. However, the ever‐expanding adoption of electronic health data makes it possible to automate this process. While relying on structured data sources such as coded problem lists or billing codes can be an efficient way to capture medical comorbidities, structured data often has poor sensitivity which can introduce bias into analytic work.^2^, ^3^, ^4^, ^5^, ^6^ Accordingly, innovative and efficient methods of analyzing free‐text documentation are crucial to realizing the electronic health record's potential to advance medical research.^7^, ^8^

One common approach to analyzing free text information is to deploy natural language processing (NLP) systems. NLP can be implemented using machine learning^9^ or human‐designed “heuristic” technologies.^10^, ^11^ Machine learning technologies are increasingly able to model non‐linear linguistic relationships and can be trained quickly on large annotated datasets. On the other hand, human‐designed heuristic‐based NLP tools are characterized by transparency (allowing for easier correction of errors) and do not require specialized high‐performing hardware such as Graphics Processing Units. Additionally, human‐designed NLP techniques can be developed using smaller annotated datasets as they incorporate their designers' knowledge of language as well as professional vernacular.^11^, ^12^ NLP has been implemented in numerous clinical applications^11^, ^12^, ^13^, ^14^, ^15^, ^16^, ^17^, ^18^, ^19^ and continues to be developed across a host of critical domains to transform natural language into data ready for computational work.

Although there have been NLP systems developed to assess for the presence of cardiovascular comorbidities in narrative electronic health data,^20^, ^21^ their portability and implementation within other health‐system databases face questions of validity.^22^ Accordingly, we sought to develop and validate NLP modules for key cardiovascular comorbidities using the longitudinal electronic health records within the Mass General Brigham system, a large tertiary care medical system in Boston, MA. Our aim was to accurately assess for the presence of major cardiovascular comorbidities—as documented by clinicians in free‐text form—in a system‐wide longitudinal health care record.

## 2 METHODS

We developed five distinct NLP modules to assess for the presence of the following cardiovascular comorbidities: (a) hypertension; (b) dyslipidemia (any subtype); (c) diabetes; (d) coronary artery disease (CAD); (e) non‐hemorrhagic stroke and transient ischemic attack (TIA). Each module was designed to assess for language that is diagnostic of these comorbidities on a phrase‐by‐phrase and sentence‐by‐sentence level. For instance, a sentence stating that, “Mrs. Smith has a history of hyperlipidemia” or one that stated, “Mrs. Smith has a history of elevated cholesterol” would be considered semantically equivalent and diagnostic of dyslipidemia. Similarly, a phrase stating, “History: uncontrolled hemoglobin A1c” would be considered diagnostic of diabetes. The ability to develop algorithms that can extract phrase and sentence‐level details to determine the presence of a diagnostic concept allow for the potential to build highly accurate NLP modules. Ultimately, the goal was to design NLP algorithms that are able to recognize phraseology that clinicians use in regular practice to represent the diagnostic concepts of interest.

For conditions where non‐binary classifications provide valuable information, we sought to develop algorithms that would be able to characterize multiple levels of clinically useful information in order to obtain granular diagnostic data. Accordingly, the modules for diabetes, CAD, and non‐hemorrhagic stroke/TIA were designed to obtain the following secondary levels of information:

1. Diabetes – (a) type 1 diabetes, (b) type 2 diabetes, (c) unspecified diabetes type.
2. CAD – (a) general CAD reference (which does not meet one of the other defined categories), (b) reference to a greater than 50% coronary stenosis, (c) unstable angina, (d) myocardial infarction, (e) ST‐segment elevation myocardial infarction, (f) coronary revascularization.
3. Non‐hemorrhagic stroke/TIA – (a) ischemic stroke, (b) embolic stroke, (c) unspecified stroke type, (d) TIA.

Designing the modules in this fashion enabled further characterization of the subtype of the diagnosis of interest as relayed through free‐text clinical documentation.

### 2.1 Document selection

**FIGURE 1:**
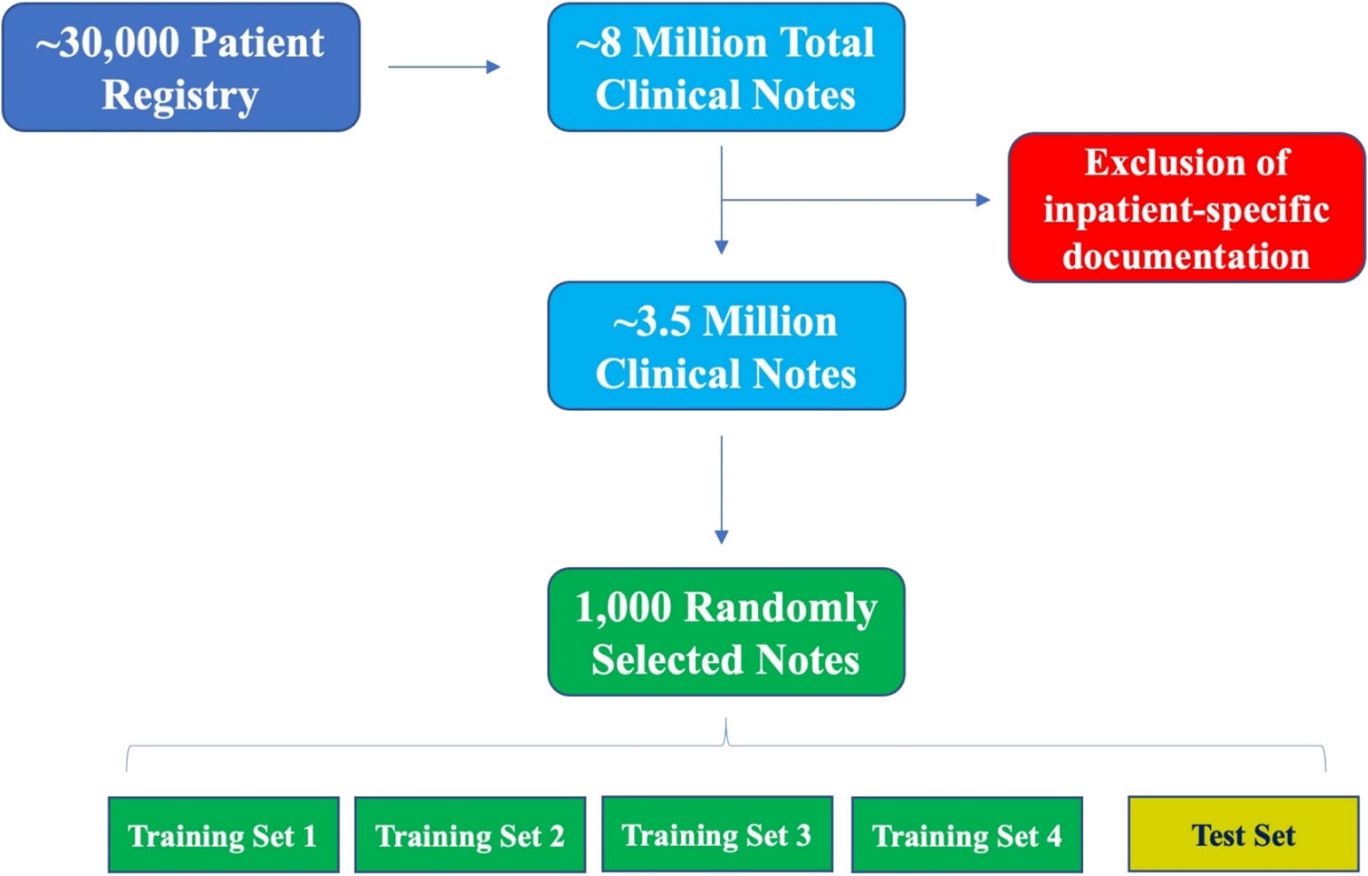
Note selection process. Schematic overview of the note selection process for manual adjudication of the five diagnostic concepts targeted for NLP development. Each of the training sets and test set contained 200 unique notes with the same proportion of outpatient and hospital discharge summaries. The NLP designer was blinded to the gold‐standard test set adjudication

Clinical notes were randomly selected out of a large, retrospective cardiovascular registry created at Brigham and Women's Hospital and Massachusetts General Hospital. The registry was comprised of ~30 000 patients who received care within the Mass General Brigham hospital system from January 2000 to July 2019. In total, the cohort generated approximately 8 million notes across all types of clinical encounters. Given the nature of the diagnostic concepts targeted for NLP development, this set of ~8 million notes was limited to predominately outpatient notes and hospital discharge summaries for further analysis. This resulted in a total of ~3.5 million notes, of which 1000 notes were randomly selected for use in NLP development. Of the 1000 notes, 800 random notes were equally divided into four “training‐sets” with the remaining 200 notes designated for the final, validation “test‐set.” See Figure 1 for a schematic of the note selection process. This study was approved by the Institutional Review Board at Mass General Brigham and was granted a waiver of informed consent.

### 2.2 Adjudication

Four internal medicine physicians at Brigham and Women's Hospital were trained to adjudicate the 1000 clinical notes for the diagnostic concepts of interest. Each physician underwent a 2‐h training session and subsequently received tailored feedback on the accuracy of their first 15 adjudicated notes prior to beginning the formal adjudication process. See the Appendix S1 for the standardized adjudication guidelines. Each of the 800 training‐set notes were adjudicated by one physician alone for all five diagnostic concepts. The final 200 notes were designated as the validation test‐set and each note was adjudicated by two physicians to optimize the accuracy of the reference standard. Agreement between the two adjudicators as measured by Cohen's Kappa in the test‐set is given in Table 1 and ranged from 0.961 to 1.00. Any adjudication discrepancies in the test‐set were resolved through a joint meeting between the two physicians to create a final validation test‐set against which the NLP software output was then compared. The physician adjudicators were not involved in the development of the NLP modules and the designer of the NLP modules was blinded to the test‐set adjudication.

**TABLE 1:** Cohen's Kappa on the adjudication of the 200 test set notes

Module	Cohen's Kappa
Hypertension	0.97
Dyslipidemia	1.00
Diabetes	0.96
Coronary artery disease	0.97
Stroke/TIA	0.98

Each physician was instructed to extract diagnostic information through a sentence‐by‐sentence review of each clinical note. Accordingly, if there were multiple sentences in a given note that referenced a history of coronary artery disease, each was logged as a positive reference to that diagnostic concept. See Table 2 for the number of unique note‐level and sentence‐level positive references for each diagnostic concept in the test set. The secure, web‐based software platform REDCap^23^, ^24^ (Research Electronic Data Capture) was used for data entry. Individual REDCap forms for each of the five diagnostic concepts were developed to facilitate information entry by the team of physician adjudicators. See the Appendix S1 for representative designs of the REDCap forms.

**TABLE 2:** Unique note‐level and sentence‐level positive references for each diagnostic concept in the 200 test set notes

Module	Note‐level references	Sentence‐level references
Hypertension	82	212
Dyslipidemia	68	169
Diabetes	29	128
Coronary artery disease	54	217
Stroke/TIA	41	168

**FIGURE 2:**
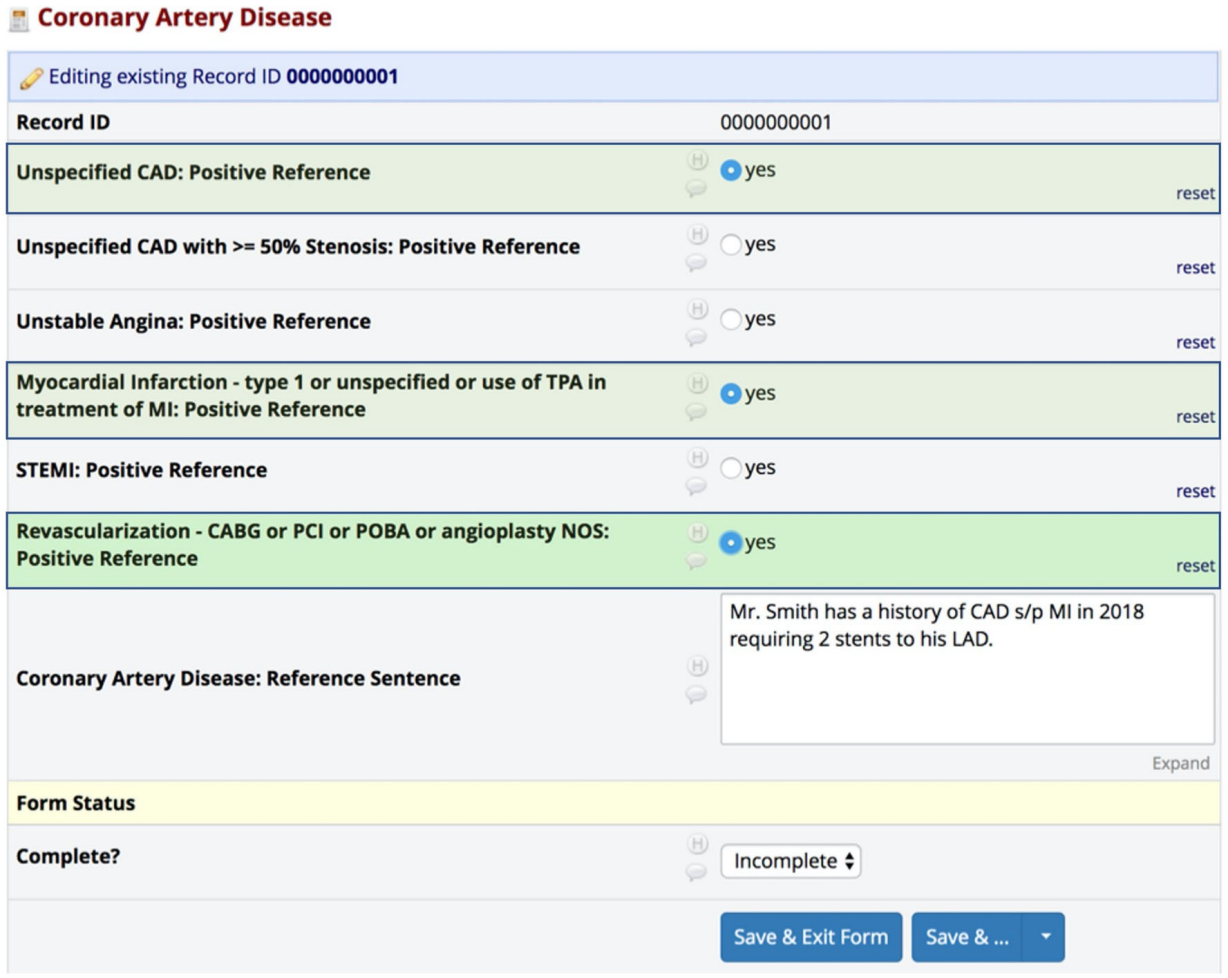
Example adjudication of multi‐layered diagnostic sentence. Example sentence and associated REDCap form of how adjudicators were instructed to input all available classification information for multi‐layered diagnostic information. In the sentence, “Mr. Smith has a history of CAD s/p MI in 2018 requiring 2 stents to his LAD,” adjudicators would click “unspecified CAD,” “myocardial infarction,” and “revascularization” to capture all available data points

When multiple levels of diagnostic information were available within a given phrase or sentence, the adjudicators were instructed to input all available classification information through the use of “radio buttons” in the REDCap forms. For instance, if a sentence stated: “Mr. Smith has a history of CAD s/p MI in 2018 requiring 2 stents to his LAD,” the adjudicators were instructed to check off the boxes for “CAD General,” “MI,” and “Revascularization” as shown in Figure 2. This process allowed for the ability to obtain detailed information from sentence‐level references and program the NLP algorithms to recognize complex and multi‐layered diagnostic concepts.

### 2.3 NLP development

NLP algorithms were created using the open‐source Canary NLP platform.^17^, ^19^, ^25^, ^26^, ^27^, ^28^ We elected to use the Canary NLP system for the following reasons: (a) it implements NLP algorithms transparently, facilitating error correction; (b) it is easily portable to other institutions and datasets; and (c) it was previously shown to achieve higher accuracy than other NLP methodologies.^28^

For each distinct NLP module, a unique set of *word classes* were created. *Word classes* contain sets of semantically‐related words that can be used to create *phrase structures*. A simplified set of *word classes* from the CAD module include:

1. >CAD ‐ cad, coronary disease, coronary heart disease, ischemic heart disease.
2. >MI ‐ acs, acute coronary syndrome, heart attack, mi, myocardial infarction, nstemi.
3. >STENT ‐ angioplasty, stents.
4. >CORONARY ‐ lad, rca, acute marginal, circumflex, lcx, left main.

**FIGURE 3:**
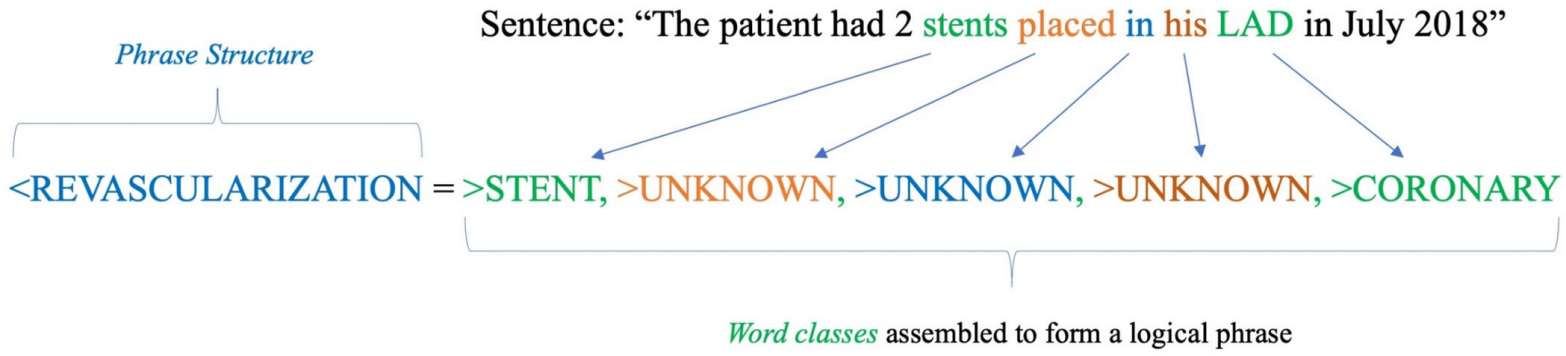
Schematic of building NLP phrase structures. Schematic of building *phrase structures* to capture diagnostic concepts using defined *word classes*. This example sentence referencing the placement of stents in a coronary artery would then resolve to an output indicating that the patient had a coronary revascularization procedure

In addition to defined *word classes*, Canary allows for the creation of an “>UNKNOWN” *word class* which accounts for sentences with undefined words. *Phrase structures* are then created from *word classes* to create meaningful units of information which can later be extracted as numbered outputs for analytic work. A *phrase structure* to capture a sentence such as, “The patient had 2 stents placed in his LAD in July 2018” is shown in Figure 3. This example sentence referencing the placement of stents in a coronary artery would then resolve to an output indicating that the patient had a coronary revascularization procedure. In the CAD module, for example, there were more than 40 distinct *word classes*, greater than 600 unique heuristic‐based *phrase structures*, and 70 numbered output types.

Each module was designed with its own unique set of *word classes* and heuristic‐based *phrase structures* to maximize diagnostic accuracy. For the 800 training set notes, a rigorous iterative process was performed whereby unique and often multilayered *phrase structures* were created to capture positive references to the diagnostic concepts of interest. When the creation of additional *phrase structures* improved sensitivity but caused a decrement to the specificity of the module, the specificity of the module was favored and such heuristics were not included in the final algorithms.

In addition to capturing positive references to the desired diagnostic concepts, the NLP system was designed to exclude negations and family history. As such, sentences describing a patient's family history of ischemic heart disease or that the patient has no personal history of CAD were programmed to be ignored by the NLP system. This intentional design was used to only identify the patient's personal history of the diagnostic concept of interest across all five NLP modules.

## 3 RESULTS

For each NLP module, we calculated the following metrics on each unique sentence‐level reference: sensitivity and positive predictive value (PPV). On the document level, we calculated the sensitivity, PPV, and specificity of each algorithm. In addition, we calculated the corrected sensitivity, corrected PPV, and corrected specificity for each module to account for true positive references that were identified by the NLP system but missed by the manual physician adjudication. For the three modules that contained multi‐layered outputs, we further calculated the sensitivity, specificity, and PPV of each distinct subcategory.

The performance of each of the five modules is given in Table 3. The NLP modules demonstrated robust performance for all the studied disease states, but was particularly accurate for the hypertension, dyslipidemia, and stroke modules with greater than 95% PPV for note‐level performance. For the three modules that had additional subcategories (e.g., diabetes, CAD, and stroke), the performance of each subcategory is presented in Table 4. For two of the subcategories – type I diabetes and ST‐segment elevation myocardial infarction – there were no references to these diagnostic concepts within the test set notes. Accordingly, we could not calculate the performance characteristics on these subcategories. Additionally, two subcategories, for example, references to a greater than 50% coronary stenosis and unstable angina – had 10 or fewer references and are reported separately in the Appendix S1.

**TABLE 3:** Performance characteristics of each of the five modules

Performance characteristics of each of the five modules						
**Hypertension**						
	Sensitivity		Specificity		PPV	
	Original	Corrected	Original	Corrected	Original	Corrected
Note level	97.5 (91.3–99.7)	97.5 (91.3–99.7)	99.2 (95.4–100)	99.2 (95.4–100)	98.7 (93.1–100)	98.7 (93.1–100)
Sentence level	96.2 (92.7–98.4)	96.3 (92.9–98.4)	NA	NA	98.1 (95.2–99.5)	98.1 (95.3–99.5)
**Dyslipidemia**						
	Sensitivity		Specificity		PPV	
	Original	Corrected	Original	Corrected	Original	Corrected
Note level	97.1 (89.8–99.6)	97.1 (89.9–99.7)	100 (97.2–100)	100 (97.2–100)	100 (94.6–100)	100 (94.6–100)
Sentence level	94.7 (90.1–97.5)	94.8 (90.4–97.6)	NA	NA	99.4 (96.6–100)	99.4 (96.6–100)
**Diabetes mellitus**						
	Sensitivity		Specificity		PPV	
	Original	Corrected	Original	Corrected	Original	Corrected
Note level	100 (88.1–100)	100 (88.1–100)	98.2 (95.0–99.6)	98.2 (95.0–99.6)	90.6 (75.0–98.0)	90.6 (75.0–98.0)
Sentence level	90.6 (84.2–95.1)	90.8 (84.4–95.1)	NA	NA	95.1 (89.6–98.2)	95.2 (89.8–98.2)
**Coronary artery disease**						
	Sensitivity		Specificity		PPV	
	Original	Corrected	Original	Corrected	Original	Corrected
Note level	98.2 (90.1–100)	98.2 (90.1–100)	94.5 (89.5–97.6)	94.5 (89.5–97.6)	86.9 (75.8–94.2)	86.9 (75.8–94.2)
Sentence level	88.5 (83.5–92.4)	88.7 (83.8–92.5)	NA	NA	93.2 (88.9–96.2)	93.3 (89.1–96.3)
**Stroke/TIA**						
	Sensitivity		Specificity		PPV	
	Original	Corrected	Original	Corrected	Original	Corrected
Note level	95.1 (83.5–99.4)	95.1 (83.5–99.4)	98.7 (95.5–99.8)	98.7 (95.5–99.8)	95.1 (83.5–99.4)	95.1 (83.5–99.4)
Sentence level	85.7 (79.5–90.6)	86.1 (80.0–90.9)	NA	NA	94.1 (89.1–97.3)	94.3 (89.4–97.4)

**TABLE 4:** Performance characteristics of NLP sub‐categories

Performance characteristics of NLP sub‐categories						
**Diabetes module: type 2 diabetes**						
	Sensitivity		Specificity		PPV	
	Original	Corrected	Original		Original	Corrected
Note level	100 (76.8–100)	100 (76.8–100)	100 (98.0–100)	100 (98.0–100)	100 (76.8–100)	100 (76.8–100)
Sentence level	96.6 (82.2–99.9)	96.6 (82.2–99.9)	NA	NA	100 (87.7–100)	100 (87.7–100)
**Diabetes module: unspecified diabetes**						
	Sensitivity		Specificity		PPV	
	Original	Corrected	Original	Corrected	Original	Corrected
Note level	100 (85.2–100)	100 (85.2–100)	98.3 (95.1–99.6)	98.3 (95.1–99.6)	88.5 (69.8–97.6)	88.5 (69.8–97.6)
Sentence level	88 (80.0–93.6)	88.2 (80.4–93.8)	NA	NA	92.6 (85.4–97.0)	92.8 (85.7–97.0)
**CAD module: CAD unspecified**						
	Sensitivity		Specificity		PPV	
	Original	Corrected	Original	Corrected	Original	Corrected
Note level	97.9 (88.7–100)	97.9 (88.7–100)	96.7 (92.5–98.9)	96.7 (92.5–98.9)	90.2 (78.6–96.7)	90.2 (78.6–96.7)
Sentence level	85.6 (77.9–91.4)	86.1 (78.6–91.7)	NA	NA	91.8 (85.0–96.2)	92.1 (85.5–96.3)
**CAD module: MI**						
	Sensitivity		Specificity		PPV	
	Original	Corrected	Original	Corrected	Original	Corrected
Note level	82.6 (61.2–95.1)	82.6 (61.2–95.1)	98.9 (96.0–99.9)	98.9 (96.0–99.9)	90.5 (69.6–98.8)	90.5 (69.6–98.8)
Sentence level	86 (72.1–94.7)	86 (72.1–94.7)	NA	NA	92.5 (79.6–98.4)	92.5 (79.6–98.4)
**CAD module: revascularization**						
	Sensitivity		Specificity		PPV	
	Original	Corrected	Original	Corrected	Original	Corrected
Note level	95.8 (78.9–99.9)	95.8 (78.9–99.9)	98.3 (95.1–99.6)	98.3 (95.1–99.6)	88.5 (69.8–97.6)	88.5 (69.8–97.6)
Sentence level	87.8 (78.2–94.3)	87.8 (78.2–94.3)	NA	NA	94.2 (85.8–98.4)	94.2 (85.8–98.4)
**Stroke/TIA module: ischemic stroke**						
	Sensitivity		Specificity		PPV	
	Original	Corrected	Original	Corrected	Original	Corrected
Note level	95.0 (75.1–99.9)	95.2 (76.2–99.9)	98.3 (95.2–99.7)	98.3 (95.2–99.7)	86.4 (65.1–97.1)	87.0 (66.4–97.2)
Sentence level	96.7 (88.7–99.6)	96.8 (88.8–99.6)	NA	NA	88.1 (77.8–94.7)	88.2 (78.1–94.8)
**Stroke/TIA module: embolic stroke**						
	Sensitivity		Specificity		PPV	
	Original	Corrected	Original	Corrected	Original	Corrected
Note level	80.0 (44.4–97.5)	80.0 (44.4–97.5)	100.0 (98.1–100)	100.0 (98.1–100)	100.0 (63.1–100)	100.0 (63.1–100)
Sentence level	70.6 (44.0–89.7)	73.7 (48.8–90.9)	NA	NA	92.3 (64.0–99.8)	93.3 (68.1–99.8)
**Stroke/TIA module: unspecified stroke**						
	Sensitivity		Specificity		PPV	
	Original	Corrected	Original	Corrected	Original	Corrected
Note level	100.0 (86.8–100)	100.0 (87.2–100)	98.9 (95.9–99.9)	98.8 (95.9–99.9)	92.9 (76.5–99.1)	93.1 (77.2–99.2)
Sentence level	90.2 (79.8–96.3)	90.3 (80.1–96.4)	NA	NA	84.6 (73.5–92.4)	84.9 (73.9–92.5)
**Stroke/TIA module: TIA**						
	Sensitivity		Specificity		PPV	
	Original	Corrected	Original	Corrected	Original	Corrected
Note level	100.0 (59.0–100)	100.0 (59.0–100)	98.4 (95.5–99.7)	98.4 (95.5–99.7)	70.0 (34.8–93.3)	70.0 (34.8–93.3)
Sentence level	100.0 (73.5–100)	100.0 (75.3–100)	NA	NA	75.0 (47.6–92.7)	76.5 (50.1–93.2)

## 4 DISCUSSION

Through a meticulous development and validation process, we designed five highly accurate NLP modules that can be used to assess for the presence of important cardiovascular comorbidities in free‐text electronic health records. When putting our metrics in the context of other methods of extracting such data—such as using ICD billing codes—it is clear that rigorous NLP modules have the potential to significantly improve the accuracy of coding cardiovascular comorbidity data. Across all five modules, we almost always achieved sensitivity, specificity, and PPV of greater than 90%. This compares to sensitivities as low as 35% for stroke,^6^ 61% for hypertension^2^ and 57% for coronary artery disease^2^ in previously published work on the accuracy of ICD coding for the ascertainment of cardiovascular risk factors.

Unlike administrative billing codes which are coded for episodically and intermittently, our NLP modules accurately extract data from individual sentences within free‐text documentation. This allows for a significant increase in the sensitivity of extracting such data, especially for patients who have only a limited number of medical encounters. Additionally, because administrative billing codes were not designed for medical research purposes, they are subject to both miscoding and under‐coding, realities which significantly impact their validity. Our NLP modules demonstrate the power of accurately extracting data from the rich narrative of free‐text documentation that is the backbone of clinical electronic health data.

Another commonly used approach for computational analysis of text is statistical analysis, also known as machine learning. Machine learning methods can also attain high accuracy but typically result in “black box” models where reasons for categorization of a particular piece of text are not clear to an external observer. This leads to difficulties in adaptation of machine learning‐based NLP tools between different institutions that may have distinct clinical vernacular and forces development of NLP tools from scratch at every organization and for every task, consuming scarce resources and impeding progress of the field.^29^ With that in mind, in this study we pursued the approach of a more transparent, human‐designed heuristic‐based NLP technology that allows tracing of each step of text analysis as well as easy modification of NLP tools to correct errors or add new functionality. We have placed the NLP modules we have designed in the public domain.^30^ We expect that their portability and transparency will allow them to serve as the foundation for a family of cardiovascular NLP tools that could be used for population management, clinical research, and clinical trial recruitment across multiple healthcare organizations.

Additional strengths of our work include the rigorous manual adjudication process by physicians of the training and test set notes, the accuracy of our modules, and the ability of our NLP systems to extract granular data from sentence‐level documentation. Furthermore, given that the repository of notes used for both the training and test sets spanned from the years 2000–2019 within a large medical system, our NLP modules likely capture the majority of linguistic formulations used to describe the clinical diagnoses of interest.

Despite the accuracy of our modules, our NLP system has some limitations. First, because our NLP modules extract data only from narrative notation—without being able to corroborate diagnoses with primary data such as imaging or laboratory results—it cannot determine if a given sentence contains accurate or inaccurate information. Accordingly, if a clinician mistakenly documented that a given patient has a history of coronary artery disease, our systems will not be able to recognize that error. Second, although the overall accuracy of our modules was excellent, the performance of our modules on the disease subcategories (such as the type of diabetes, CAD subcategory, and type of stroke) is harder to categorize given that there was a limited number of such sub‐diagnoses present in the test set notes. Finally, because our clinical notes came from a large cardiovascular repository from two academic medical centers in the United States, the performance of our modules on other sets of documentation or those from other institutions may be different.

The accurate extraction of data from clinical records is critically important for prospective and retrospective clinical research, including for recruitment for clinical trials and for population‐based studies. As demonstrated through our work, NLP has the potential to accurately identify disease states from the electronic medical record, enabling the robust description of baseline characteristics. Our five NLP modules—specifically built to identify individuals with cardiovascular disease comorbidities—is a highly accurate and open‐source system that will allow researchers to better understand the baseline characteristics of the patients in their research cohorts.

## CONFLICT OF INTEREST

Deepak L. Bhatt discloses the following relationships ‐ Advisory Board: Cardax, Cereno Scientific, Elsevier Practice Update Cardiology, Medscape Cardiology, PhaseBio, PLx Pharma, Regado Biosciences; Board of Directors: Boston VA Research Institute, Society of Cardiovascular Patient Care, TobeSoft; Chair: American Heart Association Quality Oversight Committee; Data Monitoring Committees: Baim Institute for Clinical Research (formerly Harvard Clinical Research Institute, for the PORTICO trial, funded by St. Jude Medical, now Abbott), Cleveland Clinic (including for the ExCEED trial, funded by Edwards), Duke Clinical Research Institute, Mayo Clinic, Mount Sinai School of Medicine (for the ENVISAGE trial, funded by Daiichi Sankyo), Population Health Research Institute; Honoraria: American College of Cardiology (Senior Associate Editor, Clinical Trials and News, ACC.org; Vice‐Chair, ACC Accreditation Committee), Baim Institute for Clinical Research (formerly Harvard Clinical Research Institute; RE‐DUAL PCI clinical trial steering committee funded by Boehringer Ingelheim; AEGIS‐II executive committee funded by CSL Behring), Belvoir Publications (Editor in Chief, Harvard Heart Letter), Duke Clinical Research Institute (clinical trial steering committees, including for the PRONOUNCE trial, funded by Ferring Pharmaceuticals), HMP Global (Editor in Chief, Journal of Invasive Cardiology), Journal of the American College of Cardiology (Guest Editor; Associate Editor), Medtelligence/ReachMD (CME steering committees), Population Health Research Institute (for the COMPASS operations committee, publications committee, steering committee, and USA national co‐leader, funded by Bayer), Slack Publications (Chief Medical Editor, Cardiology Today's Intervention), Society of Cardiovascular Patient Care (Secretary/Treasurer), WebMD (CME steering committees); Other: Clinical Cardiology (Deputy Editor), NCDR‐ACTION Registry Steering Committee (Chair), VA CART Research and Publications Committee (Chair); Research Funding: Abbott, Afimmune, Amarin, Amgen, AstraZeneca, Bayer, Boehringer Ingelheim, Bristol‐Myers Squibb, Cardax, Chiesi, CSL Behring, Eisai, Ethicon, Ferring Pharmaceuticals, Forest Laboratories, Fractyl, Idorsia, Ironwood, Ischemix, Lexicon, Lilly, Medtronic, Pfizer, PhaseBio, PLx Pharma, Regeneron, Roche, Sanofi Aventis, Synaptic, The Medicines Company; Royalties: Elsevier (Editor, Cardiovascular Intervention: A Companion to Braunwald's Heart Disease); Site Co‐Investigator: Biotronik, Boston Scientific, CSI, St. Jude Medical (now Abbott), Svelte; Trustee: American College of Cardiology; Unfunded Research: FlowCo, Merck, Novo Nordisk, Takeda. Ron Blankstein received research support from Amgen Inc. and Astellas Inc. Alexander Turchin reports having equity in Brio Systems and research funding from Astra Zeneca, Eli Lilly, Edwards, Novo Nordisk and Sanofi.

## Supporting information

**AppendixS1**: Supporting Information.

## Data Availability

The data that support the findings of this study are available from the corresponding author upon reasonable request.
