# Supplementary material for: Natural language processing for the assessment of cardiovascular disease comorbidities: The cardio‐Canary comorbidity project
Source: Clin Cardiol. 2021 Aug 4;44(9):1296–304. doi: 10.1002/clc.23687 (PMC8428009; doi:10.1002/clc.23687)
Supplement: Supplementary file 1 — AppendixS1: Supporting Information. [file CLC-44-1296-s001.docx]

**SUPPLEMENTAL MATERIAL**

**Table of Contents:**

**1. Adjudication Guidelines**

**2. Representative Designs of REDCap forms**

**3. CAD module subset results: (1) greater than 50% coronary lesion and (2) unstable angina results**

**~~~~~~~~~~~~~~~~~~~~~~~~~~~~~~~~~~~~~~~~~~~~~~~~~~~~~~~~~~~~~~~~~~~~~~~~~~**

**1. Adjudication Guidelines:**

Adjudication guidelines for a positive **personal history** of a given diagnosis or event. Family history of events or diagnoses are to be ignored.

1. **Hypertension**: defined as the documentation of chronic hypertensive syndromes
   - *Note*: This includes hypertensive emergency and hypertensive urgency as documented in the chart as these events almost always present on the background of chronic hypertension. Other terms such as “hypertensive retinopathy,” “hypertensive cardiomyopathy,” etc. should also count as a positive diagnosis.
   - If the term “high blood pressure” (or similar phrase) is documented in the context of a chronic hypertensive syndrome, this should be adjudicated as a positive diagnosis of hypertension.
     - When determining if the term “hypertensive” connotes a chronic hypertension syndrome, have a high threshold to code it as such. Meaning, only if the term “hypersensitive” is flanked by other words in the direct sentence that connote that this is a chronic HTN syndrome should it be taken as a positive reference.
     - You are not tasked to look at specific (numeric) blood pressure measurements.
2. **Dyslipidemia**: defined as documentation of dyslipidemia (any subtype).
   - Terms such as hyperlipidemia, hypertriglyceridemia, high LDL, elevated cholesterol, etc., all count as positive references.
   - A specific numeric cholesterol value (e.g., documentation that LDL is 200 mg/dL) should **not** be considered a positive reference unless it is qualified by another term such as “LDL is elevated to 200 mg/dL.”
     - “LDL not at goal,” “Elevated LDL,” etc. should all be characterized as a positive reference.
3. **Diabetes**: defined as documentation of any subtype of diabetes.
   - A reference to a medication used for diabetes should only be considered as diagnostic of diabetes when there is an associated reference to HgbA1c, sugar, diabetic complications, etc.
   - A specific numeric HgbA1c value or fasting blood glucose value (e.g., documentation that A1c is 9%) should not be considered a positive reference unless it is qualified by another term such as “A1c is elevated to 9%”
     - “A1c not at goal,” “Uncontrolled A1c,” “Elevated A1c,” etc. should all be characterized as a positive reference.
   - The REDCap forms will include checkboxes for type I DM, type II DM, or unspecified.
4. **Coronary Artery Disease**: defined as a documented history of CAD, history of unstable angina, history of MI (excluding type II MI), history of STEMI, or history of revascularization (CABG, PCI, angioplasty, etc.). REDCap will include check boxes for:
   - CAD unspecified – for unspecified documentation of CAD. This includes references to left heart catheterization or imaging data that discusses or references the degree of stenosis.
     - Ex 1: “LHC in April 2010 with 40% stenosis in RCA.”
     - Ex 2: “Mr. Smith is a 60M with history of CAD, DM, HLD, etc.”
     - Ex 3: “Patient has a history of ischemic cardiomyopathy”
       - Note: ignore documentation of stress test results in the absence of clinical interpretation stating CAD.
         - *Example 1*: “Patient recently had a positive nuclear stress test” should **not** be counted as evidence of CAD.
         - *Example 2:* “Patient recently diagnosed with CAD after stress testing demonstrated anterior ischemia” should be counted as evidence of CAD given that a clinician interpreted the result.
   - CAD with > = 50% stenosis – if left heart catheterization or imaging is reported to show stenosis or lesion > = 50% (includes descriptions like “CTO,” “subtotal occlusion,” “high grade lesion,” etc.), please click the box that specifies > 50% stenosis.
   - Unstable angina: this box should be checked for references to unstable angina.
     - **Note**: if, in the immediate surrounding text (one sentence before or after), the clinician describes that there was a cardiac biomarker elevation, then code the entire set of text as an “MI” and not “unstable angina.” Example of this: “Ms. Smith presented with chest pain and her troponin was elevated. She therefore underwent PCI for unstable angina.” This entire sequence should be coded as “MI” and not “unstable angina” despite the fact that the provider referenced unstable angina.
   - MI – for either specified type 1 MI, unspecified MI, or if TPA was used as treatment for MI.
     - A type II MI or a reference to an MI due to some demand event should not be characterized as an MI. Please use the one sentence before and one sentence after the reference to adjudicate whether the reference to MI is actually a reference to a type II MI.
       - Examples: “NSTEMI – will obtain echo. Likely secondary to demand from… etc.” Or, “NSTEMI – likely due to demand from tachycardia, etc…” These should not be captured as a positive hit in our algorithm so do not code as such. Have a high threshold to do this — meaning, only ignore the reference to MI or NSTEMI if there is a very direct reference by the clinician that this was a demand event.
   - STEMI – for documented STEMI
   - Revascularization – documentation of history of CABG, PCI, or angioplasty.
5. **Ischemic** **stroke** 🡪 identified as any history of non-hemorrhagic stroke or TIA.
   - Terms that reflect cerebral infarcts on imaging should be included as a positive diagnosis. Ex: “MRI demonstrated old infarct in the right frontal lobe.”
   - REDCap will have four options:
     - Ischemic stroke – in addition to clear references to ischemic strokes, if there is a reference to a non-hemorrhagic stroke in a given brain location, code that reference as an ischemic stroke.
     - Embolic Stroke – in addition to clear references to embolic strokes, if there is a reference to an acute occlusion of a vessel, code that as a reference to an embolic stroke.
     - Stroke Unspecified – use this box for any general reference to non-hemorrhagic strokes.
     - TIA

**2. Representative Designs of REDCap forms:**

**
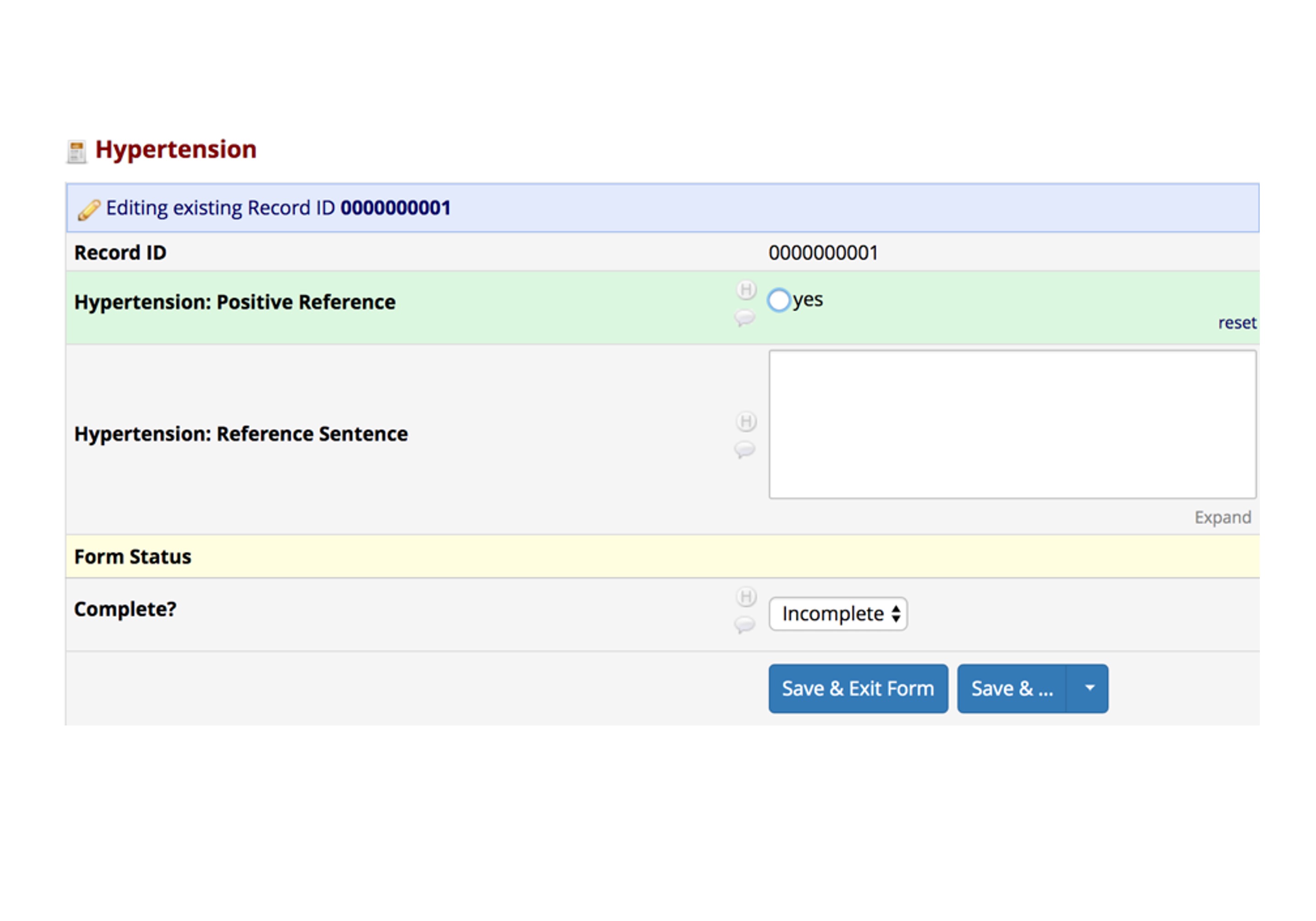
**

**
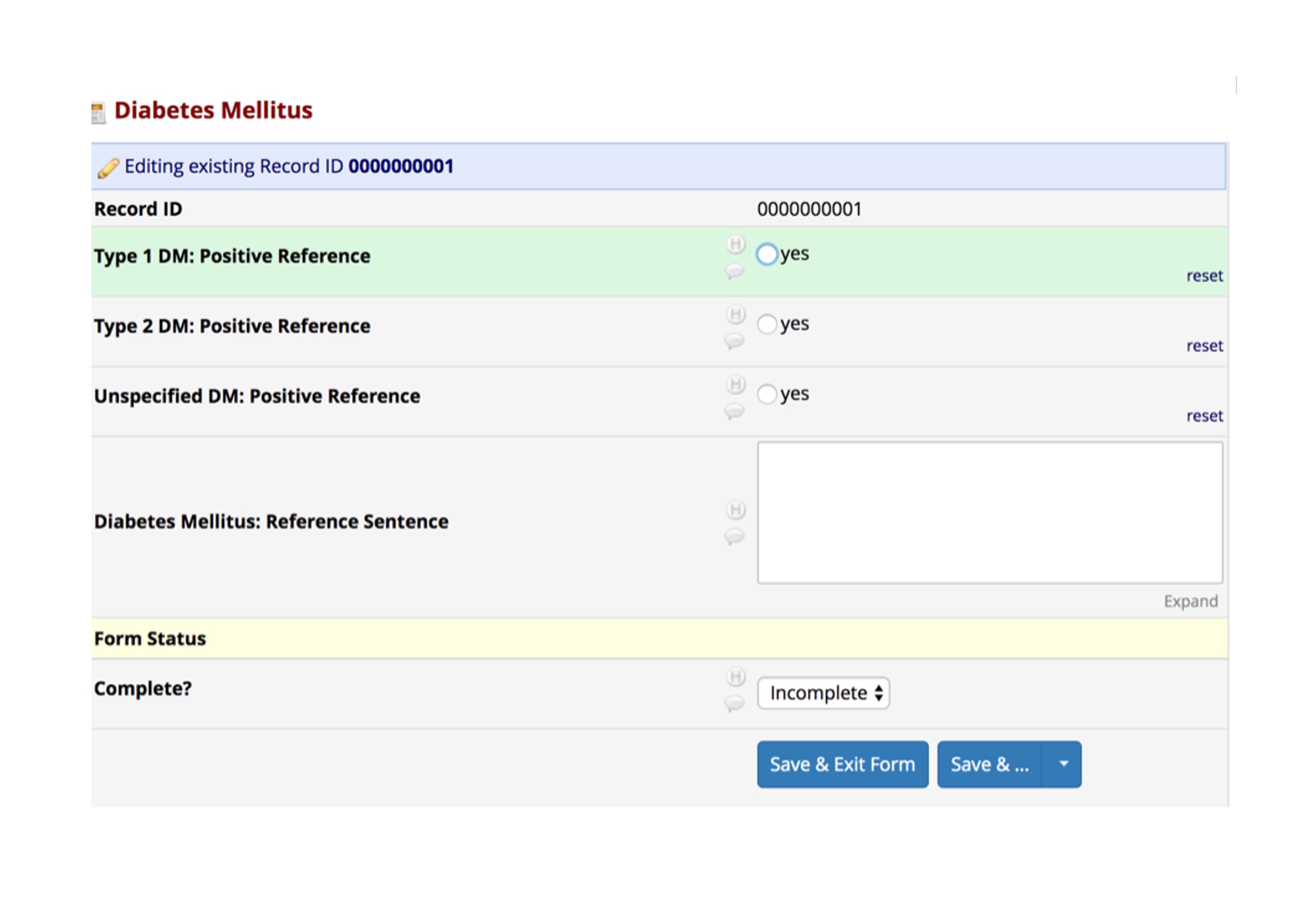
**

**3. CAD high grade stenosis and unstable angina results:** given that these two subcategories had 10 or fewer references in the test set notes, we report these results separately here

| **CAD Module:** CAD with greater than or equal to 50% coronary lesion | | | | | | |
| --- | --- | --- | --- | --- | --- | --- |
|  | Sensitivity | | Specificity | | PPV | |
|  | Original | Corrected | Original | Corrected | Original | Corrected |
| Note Level | 80  (28.4-99.5) | 80  (28.4-99.5) | 99.0  (96.3-99.0) | 99.0  (96.3-99.0) | 66.7  (22.3-95.7) | 66.7  (22.3-95.7) |
| Sentence Level | 62.5  (24.5-91.5) | 62.5  (24.5-91.5) | NA | NA | 71.4  (29.0-96.3) | 71.4  (29.0-96.3) |
| **CAD Module:** Unstable Angina | | | | | | |
|  | Sensitivity | | Specificity | | PPV | |
|  | Original | Corrected | Original | Corrected | Original | Corrected |
| Note Level | 0.0  (0.0-84.2) | 0.0  (0.0-84.2) | 100.0  (98.2-100) | 100.0  (98.2-100) | NA | NA |
| Sentence Level | 0.0  (0.0-84.2) | 0.0  (0.0-84.2) | NA | NA | NA | NA |
